# Monitoring and imaging pH in biofilms utilizing a fluorescent polymeric nanosensor

**DOI:** 10.1038/s41598-022-13518-1

**Published:** 2022-06-14

**Authors:** Charlotte Kromer, Karin Schwibbert, Ashish K. Gadicherla, Dorothea Thiele, Nithiya Nirmalananthan-Budau, Peter Laux, Ute Resch-Genger, Andreas Luch, Harald R. Tschiche

**Affiliations:** 1 Division 75 “Product Materials and Nanotechnology”, Department Chemical and Product Safety, German Federal Institute for Risk Assessment (BfR), Max-Dorn-Str. 8-10, 10589 Berlin, Germany; 2 Institute of Pharmacy, Freie Universität Berlin, 14195 Berlin, Germany; 3 Department Materials and the Environment, Federal Institute for Materials Research and Testing, 12205 Berlin, Germany; 4 Department Biological Safety, German Federal Institute for Risk Assessment, 12277 Berlin, Germany; 5 Division 1.2 “Biophotonics”, Department Analytical Chemistry, Reference Materials, Federal Institute for Materials Research and Testing (BAM), Richard-Willstaetter-Str. 11, 12489 Berlin, Germany

**Keywords:** Biosensors, Biofilms, Nanoparticles

## Abstract

Biofilms are ubiquitous in nature and in the man-made environment. Given their harmful effects on human health, an in-depth understanding of biofilms and the monitoring of their formation and growth are important. Particularly relevant for many metabolic processes and survival strategies of biofilms is their extracellular pH. However, most conventional techniques are not suited for minimally invasive pH measurements of living biofilms. Here, a fluorescent nanosensor is presented for ratiometric measurements of pH in biofilms in the range of pH 4.5–9.5 using confocal laser scanning microscopy. The nanosensor consists of biocompatible polystyrene nanoparticles loaded with pH-inert dye Nile Red and is surface functionalized with a pH-responsive fluorescein dye. Its performance was validated by fluorometrically monitoring the time-dependent changes in pH in *E. coli* biofilms after glucose inoculation at 37 °C and 4 °C. This revealed a temperature-dependent decrease in pH over a 4-h period caused by the acidifying glucose metabolism of *E. coli*. These studies demonstrate the applicability of this nanosensor to characterize the chemical microenvironment in biofilms with fluorescence methods.

## Introduction

Biofilms are consortia of microorganisms adhered to a surface and surrounded by a self-produced matrix of extracellular polymeric substances (EPS) ^1^. This matrix facilitates their survival and increases the resistance to external influences such as disinfectants ^2^. Such biofilms, that are ubiquitous both in nature and in the man-made environment, can be found on numerous surfaces, including water piping systems, food, household items, and medical devices ^3,4^.

Numerous factors can influence the formation, growth, and dispersion of biofilms and thus their harmful effects on human health, such as water and food contamination or infection ^5–8^. This includes temperature, nutrient composition, shear forces or the pH of the media in which a biofilm is formed ^9–11^. Also, these parameters can differ and change between the biofilm matrix and the environment surrounding the biofilm. Inside biofilms local microenvironments can be formed ^12^. The pH in bacterial biofilms is of central importance for many metabolic processes. For example, for dental biofilms, the pH in the extracellular matrix is the key factor for the development of dental caries ^13^. Extended periods with low pH (< 5.5) at the biofilm-tooth interface after sugar consumption can lead to slow demineralization of the underlying enamel ^14,15^. Biofilms can also induce material corrosion (termed microbially induced corrosion) which can cause damage, e.g., in power plants, refineries, petrochemical facilities, and maritime infrastructure ^16–18^. Therefore, there is a growing need to investigate chemical gradients in biofilm environments and the internal microenvironments in more detail ^19^.

The reliable measurement of extracellular pH within biofilms and the measurement of pH gradients over larger areas or longer periods of time are very challenging and tedious. Although microelectrode-based techniques are widely used in biological systems, their applicability is limited by the tip size and the small electrode area, enabling only a single point detection per measurement ^20^. For the monitoring of larger areas, the electrode must either be moved within the biofilm or multiple electrodes at different positions of the biofilm must be applied ^21^. In addition, measurements with microelectrodes are invasive and can lead to an irreversible destruction of the biofilm. Alternatively, pH can be optically determined, utilizing, e.g. fluorescence techniques such as pH-responsive molecular optical probes and pH indicators which are relatively inexpensive and easy to use ^22^. A general limitation of molecular sensors is the challenging preparation of ratiometric sensors that can account for signal fluctuations caused by fluctuations in the excitation light intensity and changes in sensor dye concentration. Moreover, many indicator dyes are taken up by bacterial cells ^23^. This could alter the sensitivity of the indicator or have a damaging effect on the bacteria as well as prevent the determination of the extracellular pH e.g. in the biofilm matrix ^24^. Moreover, molecular dyes can suffer from a relatively low photostability under microscopic conditions hampering the measurement of time-dependent pH changes. Fluorescent nanosensors can overcome some of these challenges ^25–27^. Such nanosensors commonly rely on polymer or silica nanoparticles (NP) labelled or doped with stimuli-responsive luminophores. Advantages of such systems include an increased brightness due to the large number of luminophores per particle, the relative ease of combining two dyes for the design of ratiometric sensors, and an improved photostability ^28,29^. Encapsulation of the reference and indicator dyes in the particle core can minimize their interaction with the biofilm. This approach requires a host or carrier matrix that is permeable for the target analyte in the case of encapsulated sensor molecules. Therefore, most ratiometric nanosensors are core stained with a reference dye and the functional groups at the particle surface are utilized for the covalent attachment of sensor molecules. Additionally, recognition moieties like certain bioligands can be utilized, to further enhance the selectivity of such nanosensors ^30^.

The many advantages of optical sensing schemes triggered an increasing interest in easy-to-prepare and simple-to-handle nanosensors to determine the pH in biofilms with a high accuracy and over extended periods of time. However, many nanosensors reported so far can only map very narrow pH ranges or have a relatively high aggregation tendency in biological systems, which makes them unsuitable for this task ^20,31^. Moreover, the broad application of such nanosensors for pH measurements in biofilms requires either commercial systems, which are not yet available, or at least sensor particles that can be easily prepared from commercial components without the need for an elaborate synthesis ^26,32^.

Here, a facile pH nanosensor made from commercial premanufactured aminated 100 nm polystyrene (PS) NP was developed. The PS NP were loaded with a pH-inert hydrophobic reference dye, here Nile Red (NR), via a simple swelling procedure and subsequently labelled with a commercial pH-responsive fluorescein dye ^33,34^. This design concept was also used in our previous work to fabricate oxygen nanosensors and can be realized under standard laboratory conditions ^32,33^. As a pH-responsive dye, fluorescein isothiocyanate (FITC) was selected for its pKa value of 6.5 and pH efficient interval, which optimally covers the physiological pH ranges present in *Escherichia coli* (*E. coli*) biofilms. The resulting pH nanosensor has a high colloidal and photochemical stability in aqueous dispersion and in biological media. It does not aggregate in biofilms and shows a very homogeneous distribution in the biofilm matrix. With this nanosensor, a pH range from about 4.5–9.5 can be imaged by confocal laser scanning microscopy (CLSM) as a prerequisite to visualize metabolic pH changes within a biofilm made from *E. coli*.

## Results and discussion

### Design and preparation of the pH nanosensor

To prepare the pH-responsive nanosensor, 100 nm PS NP were chosen, that are readily available in a broad size range from nm to µm and with different surface functionalizations. These PS NP are biocompatible and stable in cell culture media. Reportedly, plain and surface modified PS NP with an overall negative charge have no cytotoxic effect on *E. coli* ^35,36^. To support this assumption, a live-dead staining of the biofilm was performed after 24 h of incubation of the PS NP used for the preparation of the nanosensor (Supplementary Fig. S4). This viability assessment confirms that the incubated PS NP have no cytotoxic effect on the biofilm.

**Figure 1:**
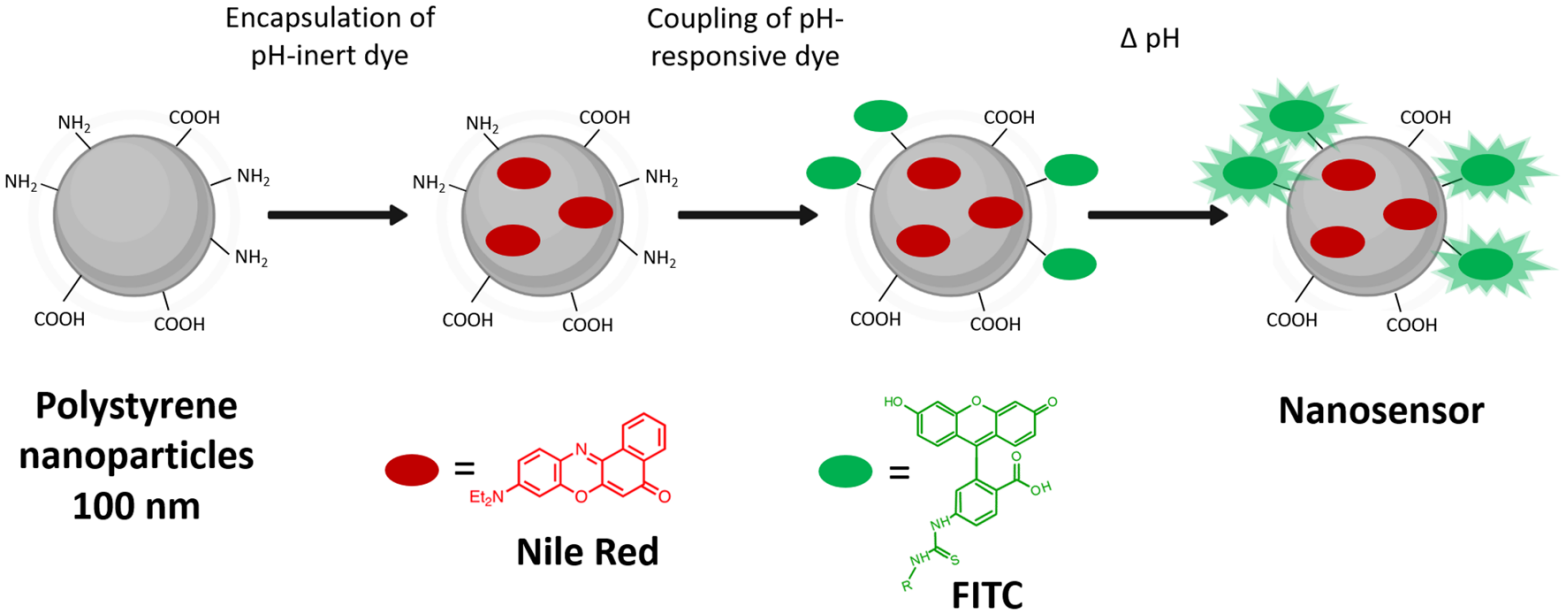
Schematic illustration of the nanosensor fabrication starting from a functionalized PS particle. NR is embedded into the particle by a swelling procedure and FITC is coupled to the PS NP by a thiourea bridge.

The simple two-step strategy for the preparation of the pH nanosensor is shown in Fig. 1. First, the reference dye NR was embedded into the PS NP by a previously established swelling method ^33^. Then, pH-responsive FITC was covalently attached to the amine groups on the PS NP surface via isothiocyanate amine coupling. Dye loading was optimized, with respect to optimal signal intensities and intensity ratios of the reference and pH-responsive dye (data not shown).

For ratiometric fluorescence sensing of pH in the visible wavelength region, hydrophobic red emissive NR was chosen as a pH-inert dye and hydrophilic and biocompatible FITC as a pH-responsive dye. NR is known to provide a homogeneous particle loading, does not show leakage from the NP in aqueous dispersions and is photochemically stable ^34,37^. FITC reveals a strong green fluorescence solely at basic and neutral pH values ^38^. The dyes exhibit spectrally discriminable emission bands as prerequisite for ratiometric sensing and can be read out with a standard CLSM setup using standard lasers and filter settings. The chosen ratiometric design concept allows a correlation of the calculated intensity ratios of the nanosensors FITC and the NR fluorescence with pH neglecting local concentration differences of the sensor. Moreover, at the chosen excitation wavelengths of 520 nm and 560 nm, no autofluorescence of the *E. coli* model biofilm was observed.

### Nanosensor characterization

**Figure 2:**
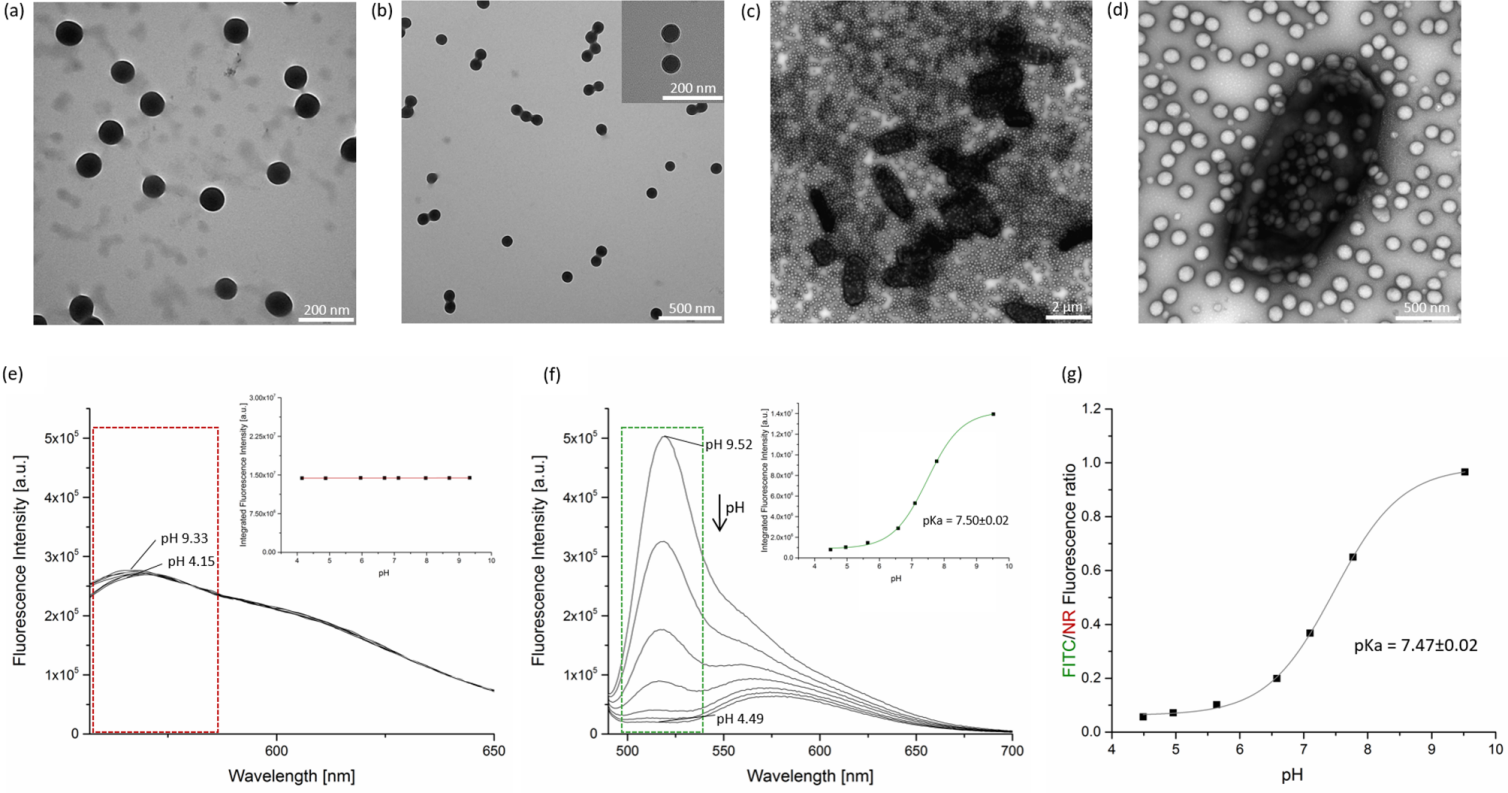
Characterization of the nanosensor. (**a**) TEM image of the precursor PS NP in water. (**b**) TEM image of the nanosensor in water. (**c**) and (**d**) TEM images of *E. coli* cells and nanosensor after 24 h incubation with 1 mg/ml nanosensor in M9 minimal medium. (**e**) Fluorescence spectra of the nanosensor excited at 530 nm (NR) in 7 buffers with different pH. Inset: Integrated FI (red box = area of signal integration) plotted against the pH value of the respective buffer. (**f**) Excited at 480 nm (FITC). Inset: Sigmoidal fit of the integrated FI (green box = area of integration) plotted against the pH value of the respective buffer. (**g**) Ratio of the integrated FI of the green FITC and the red NR emission plotted against the corresponding pH with sigmoidal curve fit.

The particle size of 100 nm provided by the manufacturer was confirmed with TEM and dynamic light scattering (DLS) (Table 1). The particle size was not altered by the introduction of NR and FITC. TEM images showed that both the PS NP and the nanosensor are monodisperse and have a spherical shape (Fig. 2a,b). The polydispersity index (PDI) assessed by DLS confirms the monodispersity of the particle suspension. Consequently, the particle size, shape, and agglomeration behavior of the NP were not affected by the dye loading and dye labelling of the NPs. The zeta potential of the PS NP before and after NR staining and FITC coupling was determined to − 30.6 ± 0.6 mV and − 38 ± 1.3 mV, respectively.

**Table 1:** Comparison of the precursor PS NP with the dye loaded nanosensor by TEM and DLS.

	Size (TEM) [nm]	Size (DLS) [nm]	PDI (DLS)	Zeta potential [mV]
PS NP	103 ± 9	133 ± 3	0.038 ± 0.023	− 30.6 ± 0.6
Nanosensor	101 ± 8	132 ± 1	0.017 ± 0.011	− 38 ± 1.3

Subsequently, the fluorescence properties of the nanosensor were investigated at different pH values in Britton-Robinson (BR-) buffer. As shown in Fig. 2 (lower panels), NR exhibits a fluorescence maximum at 560 nm upon excitation at 530 nm, while FITC shows a fluorescence maximum at 520 nm upon excitation at 480 nm at neutral and basic pH. The pH-dependent fluorescence measurements confirmed that the fluorescence intensities (FI) of NR is pH-independent (Fig. 2e) while the FI of FITC correlates with changes in pH (Fig. 2f). The FITC fluorescence signal is highest at pH 9.5, decreases upon acidification, and eventually disappears at pH ≤ 4.5. Integrating the FI of FITC in the peak area (Fig. 2f, green box) and plotting against pH reveals a sigmoidal behavior (Fig. 2f, inset), whereas that of NR remains constant (Fig. 2e, inset). The plot of the ratio of the integrated FI of FITC and NR as function of pH (Fig. 2g) yields a pKa value of 7.47 ± 0.02 that is slightly shifted to basic pH values compared to unbound FITC (pKa value of 6.5) ^39^. This shift is attributed to the coupling of FITC to a negatively charged particle ^40^. Despite the pKa shift from 6.5 to 7.5, the working range of the nanosensor of about pH 4.5–9.5 is still relatively large. Thus, the sensor is well suited for fluorometric pH sensing in the physiological pH range of *E. coli* ^41^.

### Evaluation of the nanosensor in model biofilms

Many factors can limit the functionality of a pH nanosensor in biological systems. Little is known about the interaction of NP with the EPS in the biofilm matrix ^42^. Different biomolecules, such as proteins, polysaccharides, nucleic acids or lipids can adsorb on the NP surface forming a corona-like coating ^43,44^. This can result in, e.g., particle agglomeration, shifts in the absorption or fluorescence maxima, changes in FI, and in the pH dependence of the optical properties used as readout parameters.

*Escherichia coli* were selected as the biological model system for assessing the nanosensors application potential. Although *E. coli* is a naturally occurring bacterium in the human intestine, it is the most common cause of bacterial urinary tract infections and is feared as a causative agent of blood poisoning and hospital infections ^45–47^. *Escherichia coli* forms biofilms in many environments which can be easily reproduced under laboratory conditions. Also, *E. coli* has metabolic pathways that lead to natural pH changes within the biofilm. Glucose serves as the primary energy source for *E. coli* and is converted to lactate, acetate, succinate, etc. by mixed acid fermentation ^48^. To test the metabolic activity of a lab grown *E. coli* biofilm, in a first experiment the pH shift after glucose inoculation was visualized with the pH indicator solution bromothymol blue. After 90 min, acidification of the medium surrounding the biofilm was clearly visible, confirming the suitability of *E. coli* biofilms for testing the nanosensor (Supplementary Fig. S5).

As a prerequisite for the functionality and performance studies with the ratiometric pH nanosensor, first a growth protocol for the biofilms in Ibidi slides was established, followed by an incubation protocol for the nanosensor. As criteria for the growth protocol, uniformity and reproducibility of biofilm growth, homogeneity of colonization on the slide and the biofilm thickness was chosen. Optimization of the nanosensor incubation focused on the homogeneous distribution of the NP in the biofilm and a sufficiently strong fluorescence signal for the CLSM studies, as this is essential for reliable pH measurements.

Within 24 h, the nanosensor accumulated in the biofilm but not inside the cells as revealed by CLSM experiments (see Supplementary Fig. S6). This supports the assumption that the nanosensor accumulates in the extracellular part of the biofilm. This extracellular accumulation points to previously described interactions of the NPs surface groups with the EPS in the biofilm matrix and is supported by the fact that negatively charged PS NP are not taken up by *E. coli* ^49,50^. In addition, the combined results of CSLM and TEM studies of the biofilm supernatant after 24 h of incubation indicated that the nanosensor does not tend to accumulate inside the bacteria. (Fig. 2c,d). The hydrophilicity of the nanosensor in the cell culture medium seemed to change. This is suggested by the better adhesion of the nanosensor particles to the TEM grid when applying nanoparticles dispersed in cell culture medium (Fig. 2c,d), compared to nanosensor particles dispersed in water utilizing the same particle concentration (Fig. 2a, b). Nevertheless, the nanosensor particles do not agglomerate or aggregate even after 24 h in cell culture medium. This is an advantage over previously published nanosensors, which often show a high aggregation tendency under these conditions which limits their biosensing performance ^20^.

**Figure 3:**
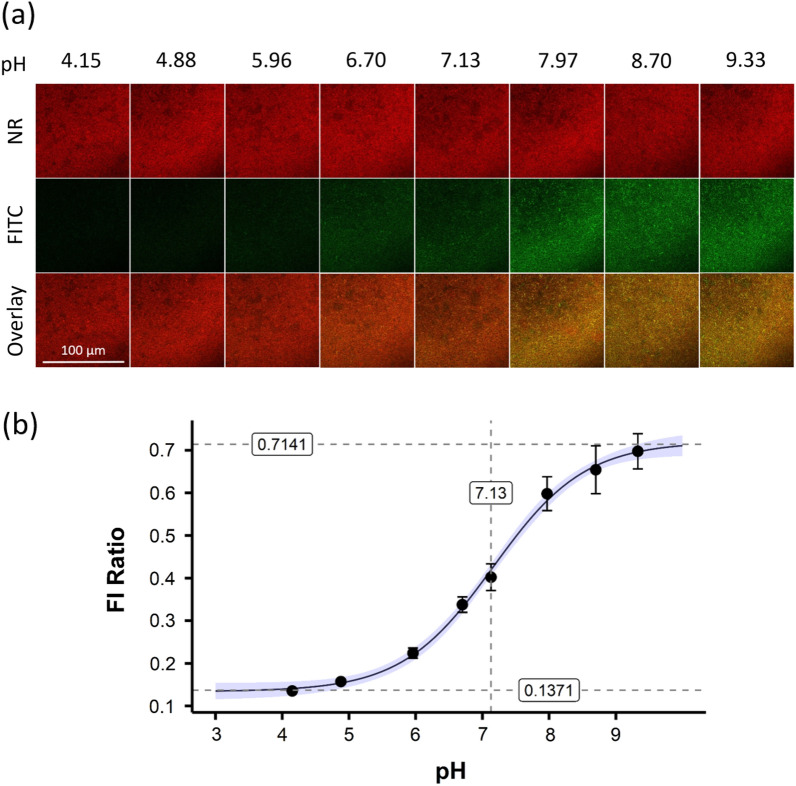
Fluorescence imaging of the nanosensor in reference buffers with CLSM. (**a**) The nanosensors FITC (green) and NR (red) fluorescence were imaged in 8 different reference buffers. For each pH value the entire depth of the biofilm was imaged as a Z-stack. A representative 2D image plus an overlay are shown for better visualization. The 100 µm scale bar applies to all images. (**b**) The FI ratio of the FITC to the NR signal was plotted against the respective pH. The blue area represents the confidence interval of the curve fit, and the error bars indicate the standard deviation. The experiments were performed as 3 independent replicates.

For the calibration of the nanosensor fluorescence inside the biofilm, the supernatant cell culture medium was replaced by a reference buffer with a well-defined pH prior to fluorescence imaging. The fluorescence signals of the nanosensor (FITC and NR) were imaged as Z-stacks at 8 different pH values (Fig. 3a). For better visualization of the FI, one representative image from each Z-stack is displayed. The yellow color of the overlayed images reflects an increased FITC signal relative to the NR signal. This ratio is highest at high pH values, here pH 9.33. The mean FI of each FITC and NR stack was calculated using the maximum intensity function for Z-stacks in ImageJ.

The FITC FI divided by the NR FI gave a FI ratio for each pH. These ratios were then plotted against the corresponding pH values (Fig. 3b). This plot was fitted with a four-parameter calibration curve to enable an inverse pH estimation from observed FI ratio values. These results confirm successful fluorometric pH sensing with the nanosensor in the pH range of about pH 4.5–9.5.

To demonstrate the potential of the nanosensor for fluorometrically imaging pH changes in active biofilms, the pH drop caused by the acidifying glucose metabolism of *E. coli* biofilms was investigated. Hence, the *E. coli* biofilms were supplemented with 10 mM glucose at 37 °C and the resulting fluorescence signals of the nanosensor were imaged over a time period of 4 h. A biofilm incubated only with the buffer but without glucose served as a control for potential non-glucose related changes in pH, e.g., due to CO_2_.

**Figure 4:**
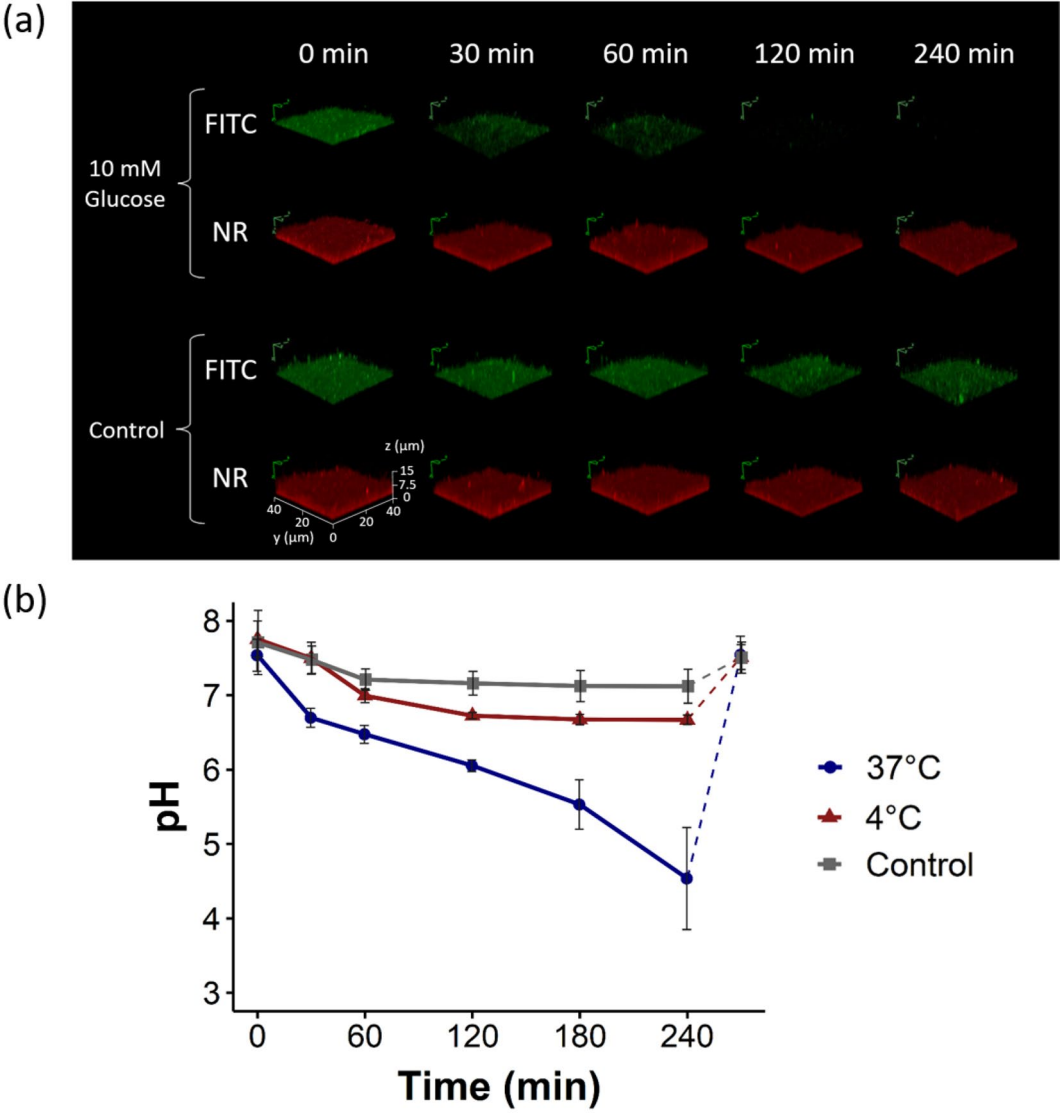
Fate of extracellular biofilm pH after glucose addition. (**a**) Z-stack CLSM images of *E. coli* biofilms after addition of 10 mM glucose (top) and control without glucose (bottom). Green = FITC signal and red = NR signal. The scale bar at the bottom right applies to all Z-stack images. (**b**) Derived pH values of the biofilm after incubation with 10 mM glucose at 37 °C, at 4 °C and the control samples without glucose. The experiments were performed as independent replicates with n = 3 and n = 6 for the control.

The nanosensor fluorescence originating from pH-responsive FITC and pH-insensitive NR was measured immediately after glucose addition (time point 0 min) and then every 30–60 min over a period of 4 h (Fig. 4a). The fluorescence of the reference dye NR did not change over time in the glucose-containing biofilm and in the control samples. Contrary, the FITC fluorescence remained constant in the control sample even after 4 h but decreased significantly in the biofilm containing glucose and eventually disappeared completely. This results in a decrease in the FITC/NR FI ratio, signaling a decrease in pH.

The pH values derived from the measured fluorescence ratios of FITC and NR with the aid of the previously acquired calibration curve are shown as a function of time in Fig. 4b. In the biofilm supplemented with glucose, the pH drops significantly from about 7.5 to about 4.5 within 4 h. In the control, no significant pH drop can be observed. Here, a maximum change from 7.5 to 7 was noticed, with no further change occurring after 1 h. When the *E. coli* biofilms are exposed to glucose at 4 °C, where their metabolism is considerably slowed down, the decrease in pH is significantly reduced compared to the studies done at 37 °C. This supports the assumption of a metabolism-induced drop in pH ^51^. To confirm that the observed decrease of the FITC fluorescence with time is not caused by photobleaching of the dye or a leaking of the nanosensor from the biofilm, the reversibility of the nanosensor was controlled. Addition of the starting buffer after 4 h restored the initial nanosensor fluorescence at pH 7.5. This shows the proper functioning of the nanosensor in terms of stability and its accumulation inside the biofilm even after long exposure times.

## Conclusion and outlook

In summary, the design, preparation, characterization, and application of a pH-responsive ratiometric nanosensor system was described utilizing commercial biocompatible polystyrene nanoparticles and the fluorescence intensity ratios of the pH-insensitive dye Nile Red, and pH-responsive FITC. The nanosensor enables fluorometric pH sensing in a range of about pH 4.5–9.5 and can monitor pH changes in biofilms over time. Thus, use of this simple nanosensor can greatly contribute to the characterization of the chemical microenvironment in biofilms.

In the future, this ratiometric pH sensor system will be used for studying other pH relevant processes in biofilms such as microbially influenced corrosion on surfaces or in depth investigations of acid stress effects in biofilms ^52^. Furthermore, pH gradients present within a biofilm could be explored by calculating the FI ratio for every single pixel in imaging experiments. Thereby, even local chemical microenvironments within biofilms could be imaged ^53^. In addition, the nanosensor can be further customized and modified as desired for specific applications, due to their ease of fabrication. For example, dyes with modified absorption or emission wavelengths and pKa values for biofilms of organism with lower pH such as acidophiles can be easily introduced ^54^. The utilization of more than two dyes as well as the combination with other imaging agents for multimodal imaging approaches is also possible.

Overall, this rational design approach for nanosensors utilizing simple and commercially available components can be beneficial for future research aimed at providing better insight into the biofilm microenvironment.

## Experimental section

### Materials and reagents

All solvents (tetrahydrofuran (THF) and ethanol (EtOH)) were of UV-spectroscopic grade, purchased from Sigma-Aldrich, and used as received. The 100 nm PS NP were purchased from Kisker Biotech and ultrasonically treated prior to use. The fluorescent dyes NR and FITC were purchased from Fluka and Sigma-Aldrich, respectively, and employed without further purification. *Escherichia coli* were purchased from DSMZ-German collection of microorganisms and cell cultures. All cell culture materials and ingredients were obtained from Sigma-Aldrich/Merck and Thermo Fisher Scientific.

### Nanosensor preparation

The nanosensor was prepared from commercially available aminated PS NP and two commercial fluorescent dye molecules. The particles had a size of 100 nm, bearing 130 nmol/mg of primary amine surface groups as determined by a Fluram assay ^55^. According to the particle manufacturer, the aminated PS NP are obtained by the reaction of carboxylated PS NP with short diamines. As this reaction is not quantitative, the PS NP bear a mixture of amine and carboxyl surface groups. The reference dye NR was incorporated into the PS NP via a swelling procedure published by Behnke et al. ^33^. In brief, NR was first dissolved in THF in a concentration of 5 × 10^−5^ mol/L. Dye loading of the PS NP was performed by addition of 100 μL of the NR-containing solution to 600 μL of an aqueous suspension of the PS NP (0.5 weight percent (w%)). After 30 min, the occasionally shaken suspension was centrifuged with an Eppendorf centrifuge 5415D at 16,000 g for 40 min. The supernatant consisting of unembedded NR dye was removed from the accordingly separated PS NP followed by two washing steps with MilliQ water with a separation step after each washing step. Next, the covalently bound dye FITC was introduced through coupling to the amine groups at the particle surface. The NP suspension (390 nmol NH_2_ groups, 1 equiv) was diluted to 5 mg/mL with phosphate buffer (0.1 M, pH 8). To this 1.5 mL suspension, 1.5 mL of a solution of FITC (2.1 µM, 5 equiv.) in PB containing 10 v% EtOH was added and shaken for 3 h with protection against light. The purification steps were the same as for the swelling procedure before, except that a total of 5 washing steps were performed and the first centrifugation/washing cycle was performed with PB containing 10 v% EtOH followed by MilliQ water.

### pH dependent fluorescence measurements

Fluorescence spectra were recorded on a calibrated spectrofluorometer (FLS920, Edinburgh Instruments). For these measurements, an integration time of 0.1 s and slit widths of 2 and 6 nm were employed for excitation and emission, respectively. For the pH dependent fluorescence behavior of the nanosensor, 2 µl of nanosensor suspension (5 mg/mL) was added to 1 mL of buffer solution. The fluorescence spectra were recorded with excitation at either 480 nm or 530 nm, with pH values of 4.49, 4.96, 5.64, 6.58, 7.10, 7.77 and 9.52, using the BR-buffer. All measurements were carried out in Hellma quartz cuvettes (QS, 10 × 10 mm^2^). The pH values of the BR-buffer solutions were adjusted with a pH meter using a glass electrode (780 pH meter, Deutsche METROHM GmbH & Co. KG) and verified with a pH meter using a InLab Micro electrode (FiveGo pH meter F2, Mettler Toledo GmbH). These pH meters were calibrated at 25 °C with standard buffers of pH = 10.01, 7.01, and 4.01 (Mettler Toledo GmbH) in three-point calibrations.

### Particle size and zeta potential

A Zetasizer (Malvern Nano ZS, Malvern Panalytical) was used to determine the zeta potential and the particle size (hydrodynamic diameter) of the nanosensor by DLS. For particle size measurement and the determination of the PDI, 2 µl of the 25 mg/ml nanosensor stock suspension was added to 1 ml MilliQ Water in a quartz glass cuvette. Thermal equilibration time was set to 60 s at 25 °C. Each intensity-weighted size distribution represents the average of ten individual DLS analyses and three independent replicates. For the determination of the zeta potential a Dip cell kit (Malvern Panalytical) was used. The NP dispersion was diluted in the same manner as done for the particle size determination. Again, the average of ten individual zeta potential analyses and three independent replicates were determined. The particle size was also assessed using a transmission electron microscope (TEM). 400 mesh 3.5 mm Formvar coated copper grids (Plano GmbH, Germany) were hydrophilized with 0.2% alcian blue (Sigma Aldrich, Germany) in 0.03% acetic acid solution. The grids were floated on alcian blue droplets for 10 min, and dried using a filter paper. The hydrophilized grids were used on the same day. 5 µl of a 5 mg/ml sample dispersion was applied on each grid, incubated for 1 min and the excess liquid was removed with a filter paper. Samples on the copper grids were observed in a Jeol 1400 Plus TEM (Jeol GmbH, Germany) operated at 120 kV. Material identification was done using diffraction pattern from published resources. Imaging was performed using a Veleta G2 camera (Olympus, Germany). Particle size was measured using iTEM software provided by Olympus. At least 4 different areas of each grid were examined per sample.

### Bacterial strain and biofilm cultivation

*Escherichia coli* TG1 DSM 6056 was used as biofilm forming microorganism ^56^. *Escherichia coli* were cultivated on Luria–Bertani (LB) medium agar plates and passaged every 3–4 weeks. For all biofilm experiments, 20 ml LB liquid medium was inoculated with single colonies and cultured overnight at 37 °C with shaking at 120 rpm on an orbital shaker (Incubating orbital shaker, Professional 3500, VWR) ^57^. The culture was diluted 1:100 in fresh LB medium and incubated for additional 1–2 h at 37 °C until cells reached the exponential growth phase. Then, 2 ml of the culture was centrifuged (2 min, 3300 g) and resuspended in 2 ml PBS. For biofilm formation, the optical density of the suspension was measured at 600 nm (Novaspec Plus, Amershan Biosciences) and adjusted to 0.01 (corresponding to approx. 1.2 × 10^6^ cells/ml) in M9 minimal medium, supplemented with 1 mM thiamine and 20 mg/L proline. For biofilm formation on the glass/liquid medium interface, 300 µl of cell suspension were then added to each well of Ibidi slides with glass bottom (8 well chamber slide, Ibidi GmbH). NP dispersions were added to a final concentration of 1 mg/ml. The slides were incubated at 37 °C on an orbital shaker, first for 60 min without shaking and then 24 h at 60 rpm for biofilm formation. Prior to imaging, biofilms were washed twice with BR-buffer to remove unbound nanosensor, and fresh buffer was added to each well.

### Imaging

All biofilms, except the 4 °C control experiments, were imaged at 37 °C in Ibidi slides using a Leica SP8 X CLSM equipped with a supercontinuum white light laser and a monochromator (Leica Microsystems). A 100 × /(N.A.1.4) objective with oil immersion was used for imaging. XY images were acquired with 2048 × 2048 or 8192 × 8192 pixels and Z-stacks in XYZ mode with 512 × 512 pixels, respectively. To obtain the Z-stacks, images were taken at 0.1 µm spacing through the biofilm. Excitation and read out emission wavelengths for FITC and NR were 480 nm and 486–525 nm and 530 nm and 537–621 nm, respectively. This choice of the emission filter settings prevents spectral crosstalk of the dyes. Biofilms without nanosensor were imaged in the same way to determine background signals and autofluorescence. The settings for imaging (laser intensity, gain, contrast etc.) were optimized in the beginning, saved, and used unchanged for all imaging procedures to establish comparability between the reference experiments and experiments for pH analysis.

### Image analysis and pH calibration in biofilm

All images were acquired with identical microscope settings. A background correction was not done as autofluorescence was not observed for the chosen measurement conditions. For the following image analysis routine with ImageJ FITC and NR Z-stacks were used. The mean FI of the entire stack was calculated using the maximum intensity function for Z-stacks. The FITC FI divided by the NR FI yields the fluorescence ratio. For referencing, a calibration curve was created using BR-buffers with 8 different pH values ranging from 4.15 to 9.33. The biofilms were washed twice with a buffer of the pH of interest and imaged with 300 µl of the buffer as supernatant. This was carried out in independent triplicates for all 8 buffers in 8 different wells. To obtain the calibration curve, a four-parameter curve was fitted to the pH calibration data using Supplementary Equation S1. Fitting was performed with a non-linear least square regression in R (V4.0.3). The fitting parameters (Supplementary Table S2) were then used to perform an inverse pH estimation from the observed value. The inverse estimation was performed in R using the *investr* package ^58^. For significance testing an unpaired two-sample *t*-test was performed.

### pH analysis in biofilms

In order to analyze changes of pH over time, glucose inoculation experiments were done. A 10 mM glucose solution in K_2_HPO_4_/KH_2_PO_4_ buffer was prepared and the pH adjusted to 9. For each well containing biofilm, buffer, and glucose, a control containing only the biofilm and buffer was imaged in the same manner. Two images were taken at each time point starting at 0 min, directly after glucose addition to the well. Then, the images of the biofilm were taken after 30, 60, 120, 180 and 240 min. After the 240-min imaging step, the biofilms were imaged for a final time to demonstrate, that the nanosensor dyes do not bleach and can be reactivated. For this final imaging step, the supernatant of the biofilm was removed and K_2_HPO_4_/KH_2_PO_4_ buffer without glucose was added to each well. For the 4 °C control experiments the microscope was cooled to 10 °C, slides were kept in the fridge at 4 °C for 30 min prior to adding the glucose solution. In between imaging the slides were kept on ice. The 3 control samples for the 37 °C and 4 °C were later combined to one control with n = 6.

## Supplementary Information

Supplementary Information.

## Data Availability

The datasets generated and/or analyzed during the current study are available in the Figshare repository 10.6084/m9.figshare.19213824.
