# Supplementary material for: Monitoring and imaging pH in biofilms utilizing a fluorescent polymeric nanosensor
Source: Sci Rep. 2022 Jun 14;12:9823. doi: 10.1038/s41598-022-13518-1 (PMC9197968; doi:10.1038/s41598-022-13518-1)
Supplement: Supplementary file 1 — Supplementary Information. [file 41598_2022_13518_MOESM1_ESM.docx]

Supplementary Information

**Monitoring and imaging pH in biofilms utilizing a fluorescent polymeric nanosensor**

Charlotte Kromer^1,2^, Karin Schwibbert^3^, Ashish K. Gadicherla^4^, Dorothea Thiele^3^, Nithiya Nirmalananthan-Budau^5^, Peter Laux^1^, Ute Resch-Genger^5^*, Andreas Luch^1,2^, Harald R. Tschiche^1^*

$${ratio}_{FI}=d+\left( \frac{a-d}{1+\left( \frac{pH}{c} \right)^{b}} \right)$$

**Supplementary Equation S1.** Curve fitting for pH calibration.

**Supplementary Table S2**. Parameters used for the inverse estimation.

| Parameter | Estimate | Std. Error |
| --- | --- | --- |
| a | 0.13383 | 0.01005 |
| b | 0.71996 | 0.01539 |
| c | 7.16552 | 0.05673 |
| d | 0.68475 | 0.05915 |

The thickness of the biofilm was determined after a growth period of 24 h and 2 washing steps with RB-buffer (**Supplementary Table S3**). For the measurements, the focus plane of the CLSM was placed at the lower end of the biofilm in BF mode and a z-stack was recorded until the minimum and maximum upper ends were reached. Three measurement points were recorded for each of the three biological replicates. After a growth period of 24 hours, the E. coli biofilms had a uniform thickness and colonized the entire Ibidi glass slide without gaps.

**Supplementary**
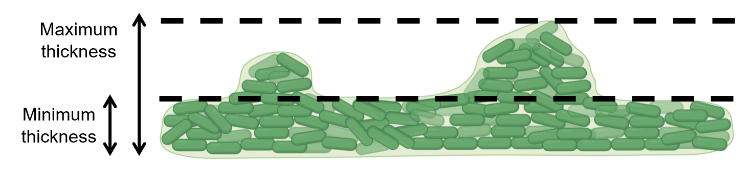
**Table S3** Minimum and maximum biofilm thickness determined with CLSM and illustration of biofilm thickness.

| Slide | Minimum thickness [µm] | Maximum thickness [µm] |
| --- | --- | --- |
| 1 | 6.45 ± 0.33 | 12.13 ± 1.35 |
| 2 | 7.82 ± 1.94 | 15.43 ± 4.43 |
| 3 | 7.88 ± 1.16 | 14.87 ± 3.28 |
| Average | 7.38 ± 0.81 | 14.14 ± 1.77 |

To visualize the structure and viability of the biofilm, a live/dead staining was performed using the BacLight kit (Thermo Fisher Scientific). **Supplementary Figure S4** shows the top and side view of a CLSM image obtained from a live/dead stained biofilm. A dense and homogeneous biofilm uniformly colonizes the slide. The high number of living bacteria (green) shows the high viability of the biofilm with is not altered when PS NP are incubated into the biofilm for 24 hours.


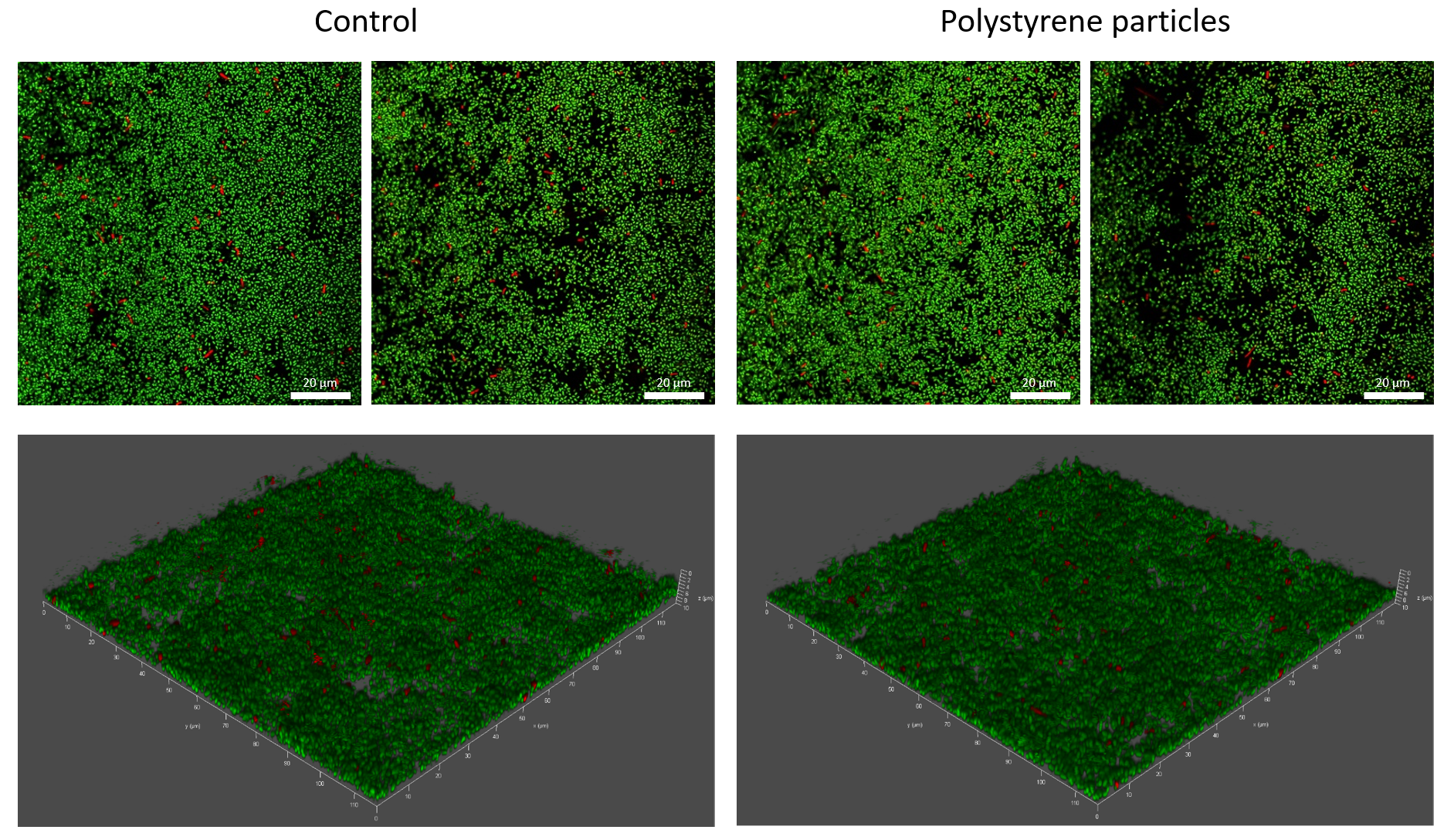
 **Supplementary Figure S4** CLSM image of a live/dead stained biofilm. The green SYTO9 stain represents living cells, the red propidium iodide stain represents dead cells. Left: biofilm grown without nanoparticles for 24 h. Right: biofilm incubated with 1 mg/ml polystyrene nanoparticles for 24 hours.


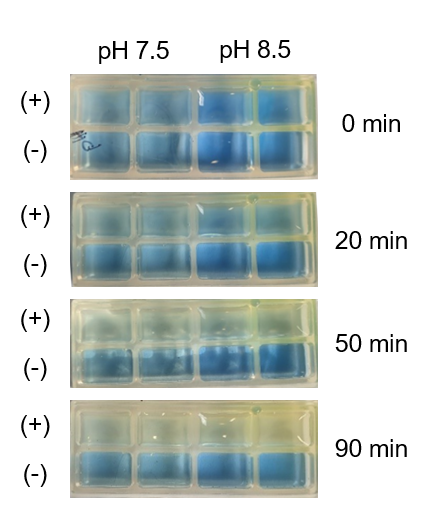


**Supplementary Figure S5** Test for metabolic activity of *E. coli* biofilms in terms of acidification of growth media. Bromothymol blue was used as pH indicator solution. The upper row samples (+) were inoculated with glucose, the lower row samples (-) were control samples without glucose. The change of color from blue to yellow, indicates acidification of the samples containing glucose.

The localisation of the nanosensors in the biofilm was assessed by incubating PS particles stained with NR but without the pH-responsive dye FITC into the biofilm. This was followed by a live staining of the biofilm with Syto9. The reason for using these particles instead of the NR-stained and FITC-labeled pH nanosensor was to avoid spectral crosstalk of the fluorescence signals from FITC and the live stain Syto9.


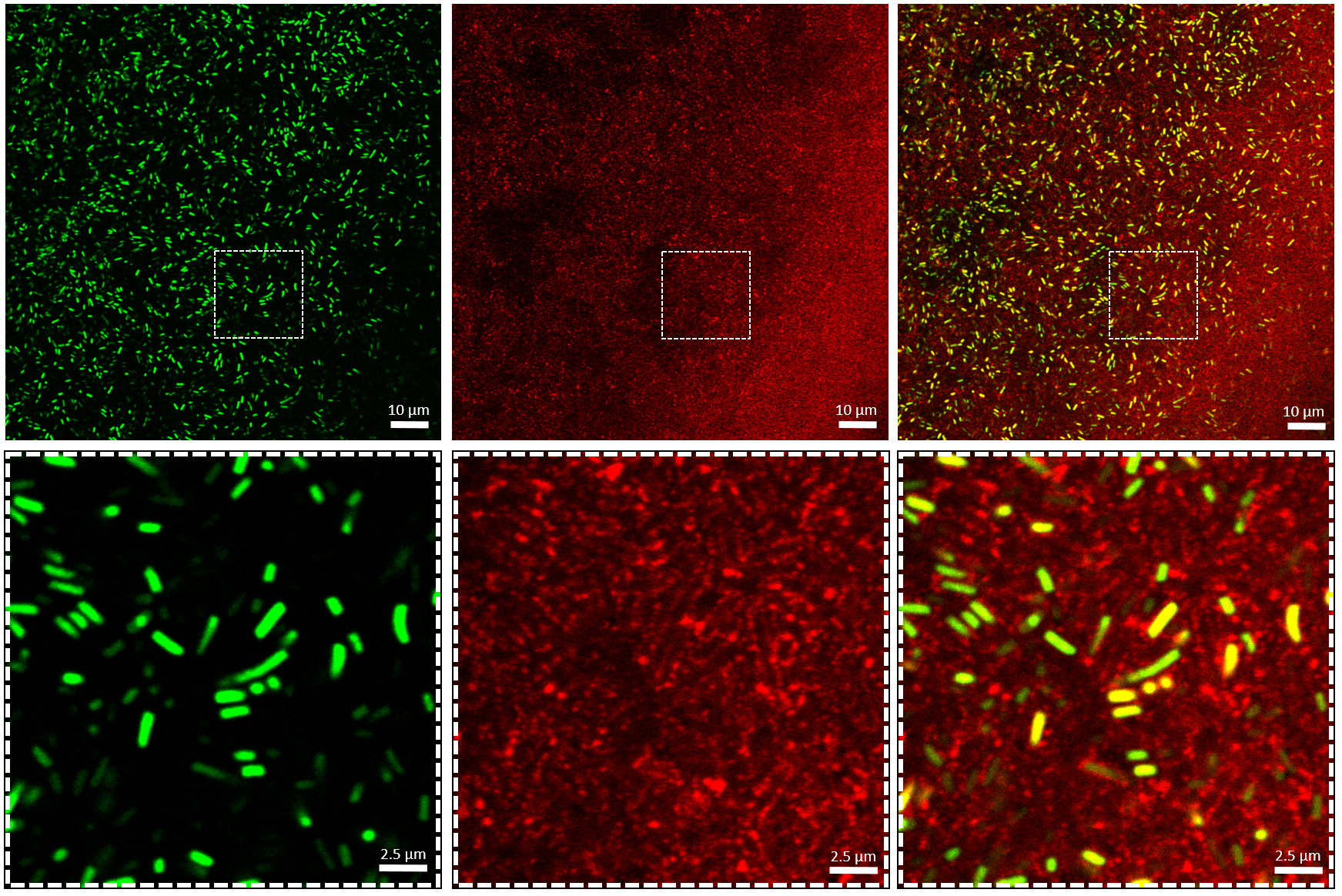
 **Supplementary Figure S6** CLSM images of the biofilm in fluorescence mode incubated with the NR-stained PS nanoparticles (red) for 24 h and subsequent live-staining of the cells with Syto9 (green). The NR fluorescence image (zoomed area, middle lower image) shows the highest fluorescence signals in extracellular regions, indicating that the nanoparticles are not taken up by the bacteria.
